# What’s New in Arrhythmogenic Cardiomyopathies

**DOI:** 10.3390/jcm11164764

**Published:** 2022-08-15

**Authors:** Tolga Çimen, Ardan M. Saguner

**Affiliations:** Department of Cardiology, University Heart Center, University Hospital Zurich, 8091 Zurich, Switzerland

Arrhythmogenic Cardiomyopathy (ACM) is a hereditary cardiomyopathy often presenting with sudden cardiac death (SCD) in young athletic individuals. Since it is a hereditary disease and alters the structural and electrical function of the heart, it is the focus of a large variety of scientific studies. The potpourri of clinical scenarios makes the name “arrhythmogenic cardiomyopathy” an umbrella term for different clinical conditions being characterized by structural cardiomyopathic changes and arrhythmias, mostly of ventricular nature.

Arrhythmogenic left ventricular cardiomyopathy (ALVC) is a distinct phenotypic form of ACM that has recently been described. It is characterized by predominantly left ventricular involvement with no or little right ventricular anomalies. In this type, several disease-causing genes have been discovered including Desmoplakin (*DSP*), Filamin C (*FLNC*), Phospholamban (*PLN*), and Desmin (*DES*) [1]. *DSP*, which accounted for half of the published cases, was the disease-causing gene with the highest frequency, followed by FLNC. An examination of the clinical characteristics of ALVC patients reveals significant electrical instability, which frequently necessitated an ICD implant (26% of all cases). Most prevalent characteristic electrocardiogram (ECG) abnormalities were low peripheral QRS voltage and negative T waves in the lateral and inferior leads (58% of all ALVC patients). Additionally, episodes of myocarditis were often observed (15% of all ALVC patients). To better estimate the risk of SCD, compared to the 2015 International Task Force Consensus (ITFC) statement and the Heart Rhythm Society (HRS) criteria, the novel arrhythmogenic right ventricular cardiomyopathy (ARVC) ventricular arrhythmia (VAs) risk prediction model developed by Cadrin-Tourigny et al. was found to be more reliable at predicting sustained VAs in a primary prevention setting [2]. This promising model has earned significant attention (Altmetric score of 174, 110 citations [including 44 in 2020 and 52 in 2021]) and the associated online risk calculator (ARVCrisk.com (accessed on 1 July 2022)) has been accessed >20,000 times illustrating that it responds to an unmet clinical need. Other independent groups have recently validated this risk calculator in their cohorts as recently carried out in a Chinese ARVC Cohort published by Zhang N et al. This rigorously conducted validation study published in this journal showed a good fitness for the prediction of arrhythmic risk [3]. The ARVC risk model in this population performed particularly well for patients undergoing ICD implantation for primary prevention. For the secondary prevention Chinese ICD population, only after recalibrating the baseline survival probability this model could be successfully applied.

According to the publication by Roudijk RW et al. clinicians should also consider 12-lead ECG indicators for the estimation of ventricular arrhythmic events in ACM patients. As highlighted by the authors one of those parameters, quantitative fragmented QRS (Q-fQRS), which is calculated by finding the number of positive and negative deflections in the QRS complexes on a 12-lead ECG, can be a warning sign of early-stage disease [4]. Even ACM family members who carried the pathogenic variant but without any clinical ACM symptoms showed a substantial elevation of Q-fQRS in comparison to controls. In addition, the most significant increase in Q-fQRS seems to occur early during the ACM disease process, which supports its potential diagnostic value in the concealed stage. In their study, pathogenic variant carriers had also higher initial Q-fQRS scores when they manifested definite ACM during follow-up.

Skin abnormalities may aid for the diagnosis of ACM in specific patient groups in addition to ECG measurements and other clinical variables. In a recent innovative study by Cabrera-Borrego E et al. published in this journal, heterozygous patients with ACM and *DSP* truncation type variants exhibited lower skin temperatures and higher transepidermal water loss, consistent microscopic cutaneous findings, and hair shaft involvement (pseudomonilethrix) with distinctive patterns such as the “fingerprint sign” [5]. The authors of this intriguing study have to be congratulated for their innovative multidisciplinary study. If their results are validated in other cohorts, skin and hair may be used as a marker of desmoplakin cardiomyopathy even in the absence of marked cardiocutaneous disease.

An exciting translational study on Bone Marrow Mesenchymal Stromal Cells (MSCs) by Scalco A et al. raised the possibility that multicellular involvement may play a significant role in the pathophysiology of ACM [6]. The examination of MSCs in the bone marrow from Desmoglein-2 (*DSG2*) mutant mice showed that these cells are more mobile and ready to mobilize to the injured region of the affected heart. This could be an important factor for myocardial remodeling and the development of fibro-fatty lesions in patients with ACM, as well as a novel approach to halting the progression of heart failure in ACM. Furthermore, cardiomyocytes derived from human induced pluripotent stem cells (hiPSC-CMs) have proven to be an effective In-vitro model for studying cardiac disease and have the potential to find possible therapeutic targets. A recently published study by Hawthorne RN et al. presented a hiPSC-CM model of ARVC obtained from a patient with a new pathogenic *DSG2* variant (*c.2358del* (p.Asp787fs)), and showed an unique phenotype with altered *DSG2* expression, increased inflammatory signaling and electrophysiological abnormalities in comparison to controls [7]. Patient-specific hiPSC experiments may enable researchers to discover specific pathophysiological pathways and assist to develop more precisely targeted therapies for various pathogenic situations including ACM.

On the other hand, it has been suggested that discontinuing competitive sports can slow the course of heart failure and lower arrhythmic occurrences in patients with ACM. In a recent study from Koch K et al., the exercise performance of ACM patients who did not discontinue with sports activity (the non-adherent group) regressed, and the left ventricular ejection fraction declined during the long-term, whereas both parameters remained stable in patients who stopped exercise activity (adherent group) [8]. Based on these findings, the authors concluded that all ACM patients, whether athletes or non-athletes at baseline, should strictly adhere to the advice to stop intense sports activity in order to slow the progression of their condition. Physical exercise has been found to be a common environmental factor in causing an arrhythmic phenotype in ACM despite heterogeneity in age, gender, underlying genotype, and other clinical traits [9]. Exercise is a significant reversible risk factor for different extents of cardiac chamber involvement, delayed versus early presentation, and variations in arrhythmic course, even among individuals with the same genotype. Another critical aspect of stopping exercise is distinguishing between the athlete’s heart and the ARVC by observing normalization of the structural changes in the athlete’s heart (detraining). In addition to this time-consuming measure, new data showed that compared to athletes, ARVC patients had considerably larger right atrial (RA), but smaller left atrial (LA) dimensions, resulting in a higher RA volume index/LA volume index ratio [10]. Together with dimensions of the right ventricular outflow tract measured in the parasternal short and long axis by transthoracic echocardiography, tricuspid annular motion, T-wave inversions, depolarization abnormalities on 12 lead ECG and serum NT-proBNP, Rossi V. et al. offered a novel diagnostic model with good diagnostic accuracy.

ACM patients are more susceptible to atrial arrhythmias due to atrial remodeling, atrial myopathy, and electrophysiological alterations at the cellular level that arise during the disease course. Although many atrial arrhythmias can be successfully treated with well-known catheter ablation (CA) techniques, there is a lack of evidence regarding the effectiveness and safety of such procedures in ACM patients. The most recent and largest systematic evaluation of the effectiveness and safety of CA for treating atrial arrhythmias in patients with ACM concluded that CA was a successful strategy for treating atrial arrhythmias including atrial tachycardias (AT), atrial fibrillation (AF) and typical atrial flutters in patients with ACM with low complication rates [11]. During a median follow-up period of 27 (13–67) months, freedom from any atrial arrhythmia recurrence after a single procedure at 12 months was reported as 74% for AF, 80% for AT and 89% for atrial flutter. One major complication (2.7%; PV stenosis requiring PV stenting) occurred. Hence, this study by Gasperetti et al. showed that CA is a promising and safe therapeutic option for atrial arrhythmias in patients with ACM, with overall long-term success rates comparable to those observed in the general population.

Psychosocial stress (PSS) is another well-known risk factor but has lately received attention in the field of ACM [12]. PSS may play a role as an environmental factor to promote disease penetration as an underappreciated risk factor in ACM. In the study by Agrimi J et al., this was unmasked by an excessive mortality of *DSG2* mutant mice under emotional stress, but also deterioration of heart function, fibrosis, and adverse remodeling in surviving mice harboring this *DSG2* mutation. Elucidating the pathogenic role of PSS as an environmental factor on patients with ACM in future studies may be important to tackle this challenging disease. In summary, this editorial summarizing recent important contributions published in the *Journal of Clinical Medicine* sheds light on the various aspects of translational and clinical research in ACM, from etiology and pathophysiological mechanisms to risk stratification, disease prevention and therapeutic potentials.
